# Association of mortality and physician experience in prehospital anaesthesia: a registry study on new physicians in Finnish helicopter emergency medical services

**DOI:** 10.1186/s13049-025-01412-4

**Published:** 2025-05-30

**Authors:** Anssi Saviluoto, Piritta Setälä, Miretta Tommila, Jussi Pirneskoski, Lasse Raatiniemi, Jouni Nurmi

**Affiliations:** 1 Emergency Medicine and Services, Helsinki University Hospital and University of Helsinki, FinnHEMS 10, Vesikuja 9, Helsinki, Vantaa, 01530 Finland; 2 Centre for Prehospital Emergency Care, Helicopter Emergency Medical Services, Tampere University Hospital, Tampere, Finland; 3 Department of Perioperative Services, Intensive Care Medicine and Pain Management, Turku University Hospital and University of Turku, Turku, Finland; 4 Research Group of Surgery, Anaesthesiology and Intensive Care, Medical Research Centre, University of Oulu, Oulu, Finland; 5 Division for Prehospital Emergency Medicine, Oulu University Hospital, Oulu, Finland

**Keywords:** Air ambulances, Emergency medical services, Learning, Prehospital anaesthesia, Intubation, Mortality

## Abstract

**Background:**

Prehospital anaesthesia is a challenging procedure, and the outcome depends on the quality of the process. Hospital-acquired anaesthesia experience does not necessarily translate to high performance in the prehospital setting. We aimed to assess the quality and practice patterns in prehospital anaesthesia related to cumulative experience amongst new prehospital critical care physicians. In this study, we aimed to evaluate whether quality indicators for prehospital anaesthesia and related mortality improve as new prehospital critical care physicians become more experienced with this intervention.

**Methods:**

We conducted a registry-based observational study including all patients who underwent anaesthesia and airway management by physicians who started working in the national HEMS between January 2013 and August 2019. Patients were grouped and compared based on the provider’s cumulative case volume at the time of the mission: 1–10, 11–20, 21–40, 41–80 and > 80 cases. The association between cumulative experience and 30-day mortality was assessed using multivariate logistic regression analysis. Secondary outcomes included first-pass intubation success, post-intubation hypoxia and hypotension, the combined use of a neuromuscular blocking agent and anaesthetic, on-scene time, mechanical ventilation usage, and rates of normocapnia, hypoxia, and hypotension at handover.

**Results:**

1,638 patients (median age 59, 64% male) were treated by 32 physicians. Median on-scene time decreased with increasing experience from 33 (interquartile range [IQR] 23–44) to 28 (IQR 19–38) minutes, *P* = 0.03.

Higher experience was associated with increased use of mechanical ventilation (*P* < 0.001) and a combination of neuromuscular blocking agents and anaesthetics (*P* = 0.03). Other secondary outcomes did not show a statistically significant difference between the groups. Crude mortality decreased from 38 to 26% in the lowest to highest experience groups. In the multivariate logistic regression analysis, the same trend was still seen with the odds ratio of the highest experience group for 30-day mortality 0.59 (95% CI 0.38–0.94, lowest experience group as a reference).

**Conclusions:**

In a prehospital critical care service, outcomes improve after a high number of prehospital cases, even when physicians with a solid foundation in in-hospital anaesthesia are employed. Limiting physician turnover may improve the quality of care.

**Supplementary Information:**

The online version contains supplementary material available at 10.1186/s13049-025-01412-4.

## Background

Prehospital anaesthesia is a critical and challenging intervention in prehospital critical care. The outcome depends not only on successful endotracheal intubation but also on the overall anaesthetic management of patients in a dynamic and unpredictable environment with changing prehospital teams. Many of these patients are in critical condition, requiring urgent hospital treatment. Inadequately performed sedation and intubation attempts have been associated with higher mortality rates in patients with traumatic brain injury (TBI) [1]. In contrast, prehospital anaesthesia by an expert provider may improve functional outcomes [2, 3].

In a previous paper, we showed that frequent repetitions in prehospital anaesthesia were associated with lower mortality, even among experienced anaesthesiologists [4]. This finding underscores that prehospital critical care should be regarded as one of several subspecialties that benefit from centralising care to experts in this specific field [5, 6]. Nevertheless, previous studies do not address what is necessary to attain a high level of proficiency in prehospital advanced airway management (PHAAM).

The learning curves for tracheal intubation and other technical procedures by novice providers have been studied in controlled settings [7]. However, skill development in emergency airway management remains a debated yet understudied topic. Furthermore, managing a critically ill patient in a prehospital setting extends beyond airway management and involves a far more complex set of clinical and nontechnical skills. Greater familiarity with the prehospital setting may enhance strategic decision-making and crew resource management while also improving manual skills, which may lead to better outcomes. To our knowledge, there are no studies on how new prehospital critical care physicians acquire the necessary competencies for high-quality prehospital anaesthesia, nor on whether their learning process impacts patient outcomes. Understanding this progression could help optimise training programs for new physicians and inform staffing strategies during their onboarding phase in prehospital critical care services.

We hypothesised that new HEMS physicians improve in patient safety and efficiency as they gain prehospital experience, leading to reduced mortality and better quality anaesthesia. Our objective was to evaluate the progression of patient outcomes and anaesthesia quality among new physicians in helicopter emergency medical services (HEMS), using recently published quality indicators for PHAAM [8].

## Methods

### Study design

We performed a registry-based observational study to assess the association between the cumulative number of physicians prehospital anaesthesia cases and 30-day mortality. In our secondary analysis, we compared process and outcome-related quality indicators according to cumulative case numbers of physicians. These included the rate of rapid sequence induction for tracheal intubation, first-pass success rate (FPS) of intubation, desaturation rate during intubation, rate of hypotension during intubation, proportions of hypoxic and not normocapnic patients at handover, and on-scene time (OST). The study period was January 2013 to August 2019. This study is reported according to the STROBE statement [9].

### Setting

The Finnish HEMS is part of Finland’s publicly funded healthcare system. During the study period, it included five physician-staffed units and one paramedic-staffed unit, which respond to both medical and trauma cases. The paramedic-staffed unit operates in Finland’s sparsely populated northern part. According to national criteria, HEMS units are dispatched by emergency response centres with minimal regional differences. Emergency medical services (EMS) can directly request assistance from HEMS if deemed necessary. We have previously described the Finnish HEMS system in detail [10].

Physicians recruited for Finnish HEMS are primarily consultants or final-year resident anaesthesiologists with at least two years of experience in anaesthesia and critical care medicine, providing them with a strong background in advanced airway management before beginning work in HEMS. The selection process typically includes a suitability assessment, including testing of psychological capacity and interpersonal skills and interviews. Before independent operative practice, new physicians undergo one to two months of training, including skill stations, full-scale simulations, and on-scene senior physician support.

### Participants

We included patients undergoing prehospital anaesthesia – defined as drug-facilitated endotracheal intubation – by physicians who began prehospital work during the study period. We excluded all patients treated by a physician in HEMS during 2012 as most of these physicians likely transferred from predecessors of the national HEMS. Patients treated by the paramedic-staffed unit were not included because a mixed background would limit the generalisability to purely physician- or paramedic-based HEMS. No exclusions were made based on the indication for airway management or patient characteristics. The studied exposure was prehospital anaesthesia managed by HEMS physicians with increasing levels of experience, and these were compared to patients whose prehospital anaesthesia was managed by physicians with 10 or fewer cases in HEMS.

### Data sources

Data were collected from a national HEMS database. HEMS teams have been required to enter details on all missions into the FinnHEMS Database since 2012, promptly after mission resolution. If any airway management was performed, a separate mandatory section for PHAAM is filled out. Entered data include vital signs at specific time points: immediately after patient contact, prior to intubation, following intubation, and at the handover to the hospital. Individual physicians have a unique identification number that can be used to anonymously acquire specific details about cases they have attended. Details of the database are provided in a previous paper [10]. The data collected follows the international recommendation for data collection in prehospital critical care and airway management [10–12]. After the mission, the HEMS crew assigns each patient a category based on the presumed primary medical reason for the mission. The categories are predefined by guidelines and include the following: out-of-hospital cardiac arrest, trauma, breathing difficulties, chest pain, stroke, acute neurology (excluding stroke), psychiatry (including intoxication), obstetrics and childbirth, infection, or other [12].

The survival of the patients was followed by the National Population Registry maintained by the Digital and Population Data Services Agency. Patient classification for TBI was performed based on the national hospital discharge diagnosis registry (International Statistical Classification of Diseases S06.1–06.9). Data entry is mandatory for all patients at the time of hospital discharge. Data extraction for mortality and discharge diagnosis was based on the unique national personal identification number (PIN) given to all permanent and temporary residents of Finland.

### Statistical methods

According to the treating physician’s cumulative experience, patients were grouped as follows: 1–10, 11–20, 21–40, 41–80 and > 80 prehospital anaesthesia cases. A histogram was drafted to visually determine a suitable range for category cut-offs. This nonlinear categorised approach was chosen because the relationship between cumulative experience and mortality was likely to be non-linear. It is intuitive and seen in previous studies that the effect of a single repetition will have a greater impact in the early phase of training compared to a more advanced stage of cumulative experience [13–15]*.*

The Chi-square test was used to compare proportions between groups; the Kruskal–Wallis test was used to compare continuous variables among independent samples. Nonparametric tests were chosen because all tested continuous variables were non-normally distributed. All vital signs were analysed as continuous variables. Results are presented as a number (percentage) and median (interquartile range [IQR]) as appropriate. Missing values were excluded.

Multivariate logistic regression was used to assess the association between cumulative experience and 30-day mortality while adjusting for age, sex, patient category, time from alarm to the patient encounter, first vital signs recorded by HEMS and whether the patient was transported directly to a university hospital.

Vital signs included in the multivariate logistic regression analysis were: heart rate, systolic blood pressure, Glasgow Coma Score (GCS) and peripheral blood oxygenation saturation (SpO_2_). Cases with missing values for any of the covariates were excluded from this analysis. Model fit was evaluated using the Hosmer–Lemeshow test and Nagelkerke’s R^2^. We conducted three sensitivity analyses, the rationale and results of which are presented in Supplement 1. This supplement also describes minor transformations applied to certain variables to facilitate analyses.

All statistical tests were performed using SPSS version 27 for Mac (IBM, Armonk, New York, USA) and visualisations produced with GraphPad Prism version 9.0 for Mac (GraphPad Software, San Diego, California USA).

## Results

**Fig. 1:**
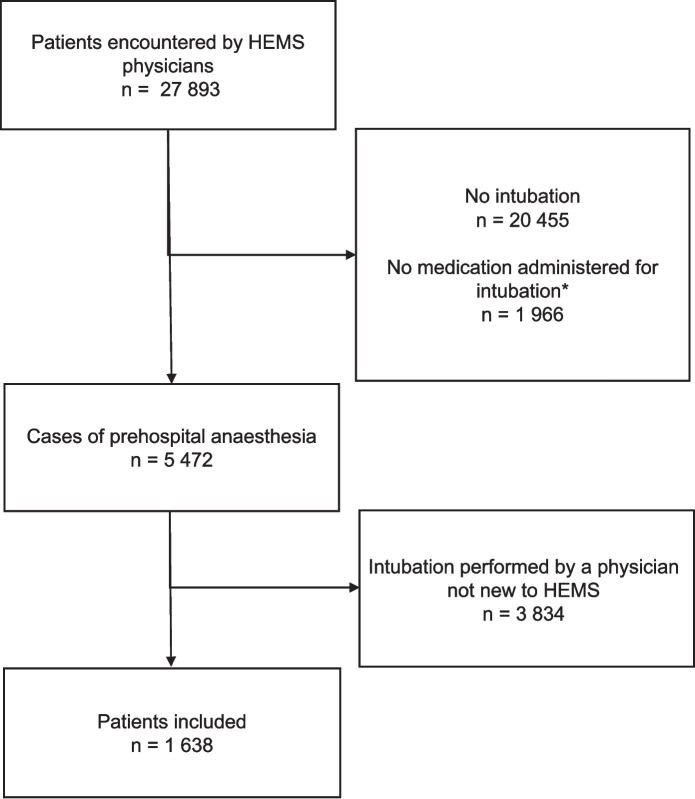
Flow chart of the patient selection. HEMS: Helicopter Emergency Medical Services. *Includes patients in cardiac arrest during intubation

During the study, 32 physicians started working in the national HEMS service, providing prehospital anaesthesia to 1,638 patients (30% of all prehospital anaesthesia cases by Finnish HEMS during the study period). The patient selection flow chart is in Fig. 1.

### Patient characteristics

The patients’ median age was 59 (37–71), of which 1,045 (64%) were male. The most common patient categories were neurological (582 [36%]), trauma (413 [25%]), intoxication (247 [15%]) and out-of-hospital cardiac arrest (229 [14%]). Other patients formed 167 [10%] cases of the study population. Median GCS was 4 (IQR 3–7) when encountered by the HEMS team, and 34 patients were sedated before HEMS arrival. Median and IQR for GCS remained constant whether these patients were analysed as missing data or as a GCS of 3. A separate online supplement shows missing data for all reported variables (Supplement 3A). Patients’ baseline characteristics according to cumulative physician experience are in Table 1.

**Table 1:** Characteristics of the patients undergoing prehospital anaesthesia according to the cumulative number of cases by new physicians in helicopter emergency medical services (HEMS). Data are presented as n (%) or median (interquartile range)

	Cumulative number of prehospital anaesthesia cases by physician									
	1–10 *n* = 327		11–20 *n* = 256		21–40 *n* = 398		41–80 *n* = 405		> 80 *n* = 252	
Age, years	62	(43–73)	60	(40–70)	59	(35–71)	56	(35–70)	57	(23–71)
Sex, male	211	(65)	153	(60)	250	(63)	257	(64)	174	(69)
Patient category										
Trauma	82	(25)	59	(23)	107	(27)	92	(23)	73	(29)
Out-of-hospital cardiac arrest	48	(15)	28	(11)	51	(13)	63	(16)	39	(16)
Neurological	127	(39)	104	(41)	145	(36)	131	(32)	75	(30)
Intoxication	37	(11)	38	(15)	63	(16)	66	(16)	43	(17)
Other	33	(10)	27	(11)	32	(8)	53	(13)	22	(9)
Glasgow Coma Score	4	(3–6)	4	(3–7)	4	(3–7)	5	(3–7)	4	(3–7)
Hypotension at HEMS encounter	15	(5)	25	(10)	34	(9)	38	(10)	22	(9)
First vital signs by HEMS										
Oxygen saturation, %	96	(92–98)	97	(92–99)	97	(93–99)	96	(92–99)	97	(93–99)
Systolic blood pressure, mmHg	135	(112–170)	134	(108–164)	130	(110–160)	132	(108–156)	130	(108–164)
Heart rate, min^−1^	92	(75–115)	92	(75–119)	93	(76–111)	93	(77–112)	95	(75–115)
Interval from emergency call to HEMS team at the patient, min	24	(16–37)	24	(17–34)	23	(17–34)	22	(17–35)	25	(18–36)

### Quality indicators

**Fig. 2:**
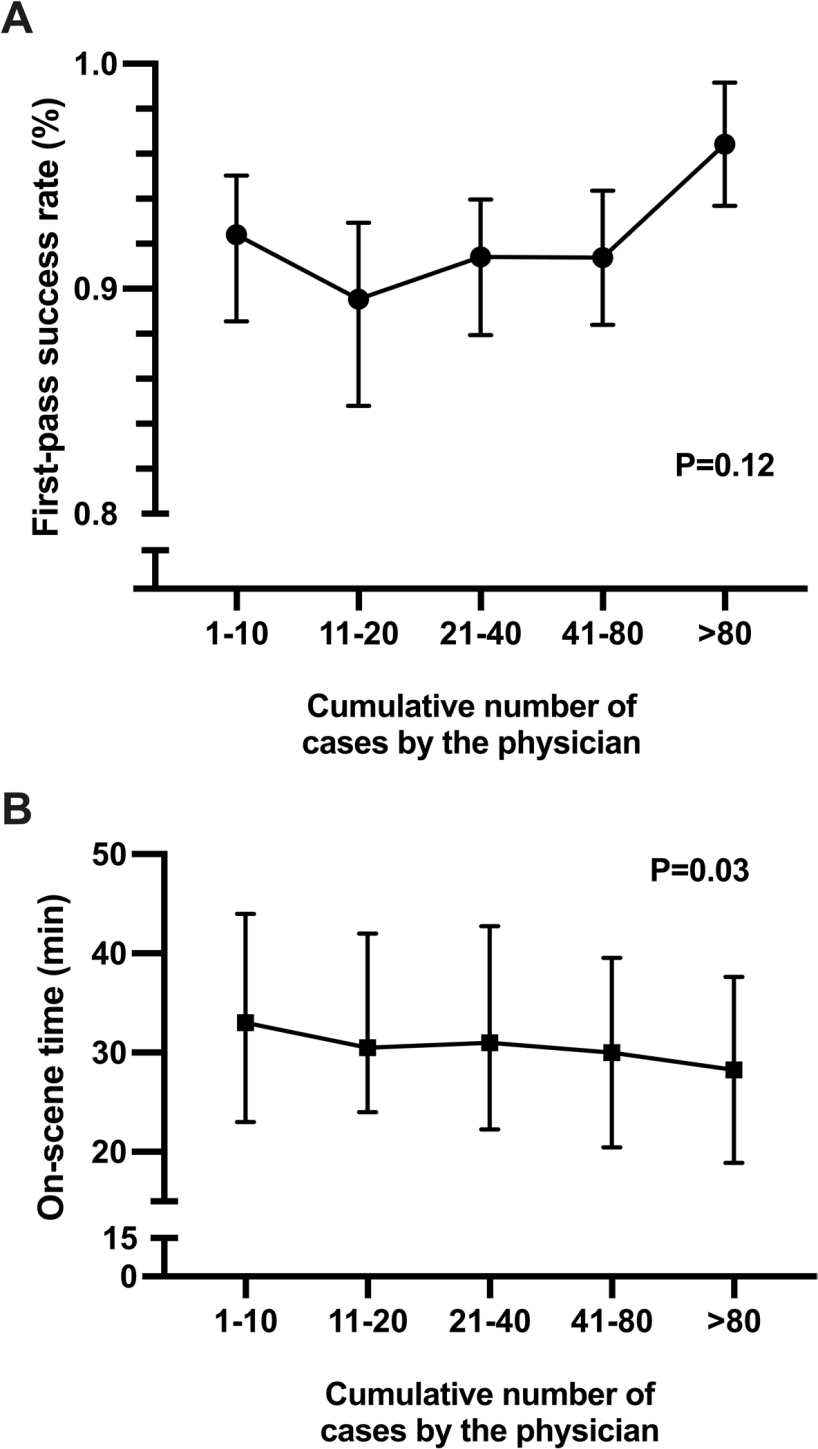
First-pass success rate of intubation (**A**) and on-scene time (**B**) of 1,638 patients undergoing prehospital anaesthesia by the treating physician’s cumulative prehospital anaesthesia case number

FPS was highest in the group with the most experience, but confidence intervals overlapped between groups (Fig. 2). OST decreased with cumulative experience (Fig. 2). The PHAAM quality indicators for each group are shown in Table 2. No difference was seen in the rates of hypoxia or hypotension after intubation or at handover to the hospital. Mechanical ventilation was more common as experience increased, but no difference could be seen in the proportion of normoventilated patients at handover. More patients received a combination of neuromuscular blocking and sedative agents in the groups with greater experience.

**Table 2:** Quality indicators of prehospital anaesthesia according to the cumulative experience of cases by new prehospital critical care physician. Rapid sequence induction is defined as use of both anaesthetic and neuromuscular blocking agent. Data are presented as n (%) or median (interquartile range). *P*-value determined using the Chi-squared-test

	Cumulative number of prehospital anaesthesia cases by physician										*P*-value
	1–10 *n* = 327		11–20 *n* = 256		21–40 *n* = 398		41–80 *n* = 405		> 80 *n* = 252		
Rapid sequence induction	299	(93)	220	(93)	320	(93)	338	(98)	173	(98)	< 0.001
Mechanical ventilation used	269	(82)	204	(80)	327	(82)	346	(85)	225	(89)	0.03
Normoventilated at handover^a^	224	(75)	181	(75)	277	(75)	275	(72)	156	(66)	0.13
Hypotension											
After intubation^b^	22	(10)	22	(11)	47	(14)	42	(13)	24	(12)	0.58
At handover^c^	15	(5)	16	(7)	23	(6)	20	(5)	17	(7)	0.55
Hypoxia											
After intubation^b^	3	(1.4)	2	(1.0)	5	(1.5)	7	(2.2)	5	(2.5)	0.75
At handover^c^	8	(2.7)	4	(1.7)	10	(2.7)	12	(3.2)	6	(2.6)	0.87

^a^EtCO_2_ 4–4.67 for patients with traumatic brain injury and 4–6 kPa for other patients. ^b^Hypotension after intubation defined as a decrease in systolic blood pressure to less than 90 mmHg or over 10% decrease from baseline whereas hypoxia is defined as a decrease below 90% or ≤ 10% drop from baseline if > 90% at time of encounter. ^c^Hypotension defined as systolic blood pressure < 90 mmHg and hypoxia defined as SpO_2_ < 90% at handover^6^

### Mortality

**Fig. 3:**
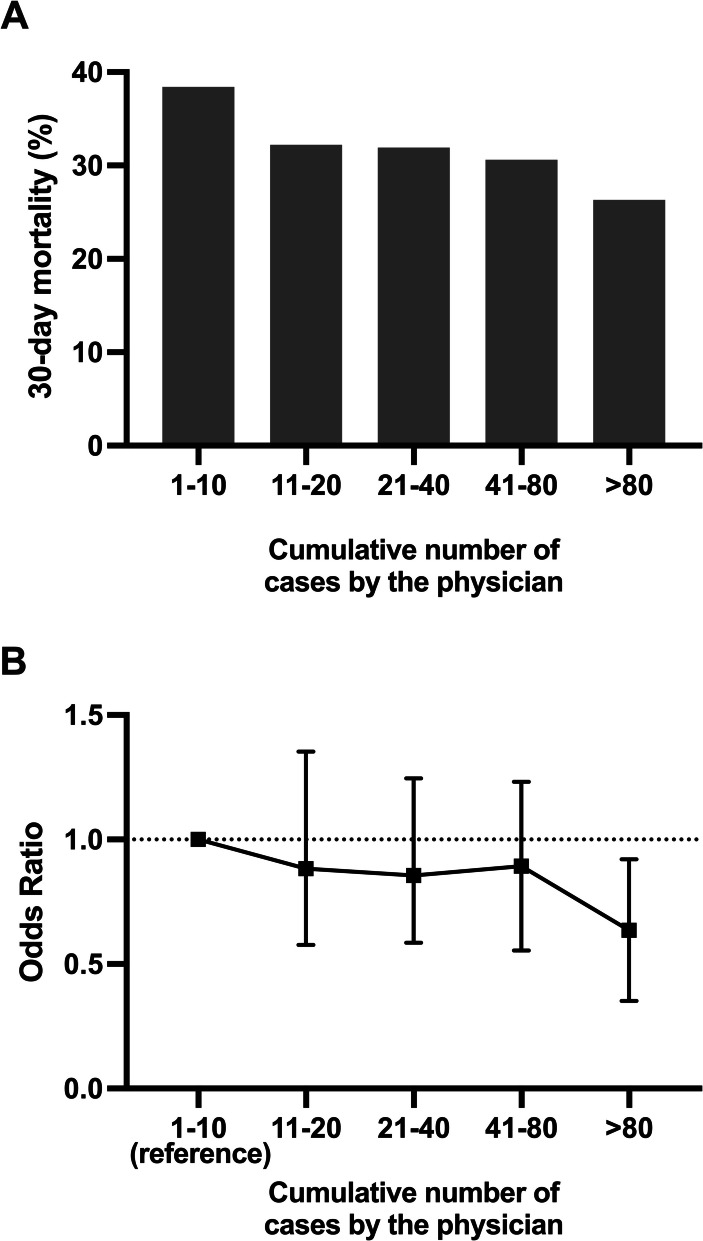
Crude 30-day mortality (**A**) and adjusted odds ratios (**B**) of 1,638 patients undergoing prehospital anaesthesia by the treating physician’s cumulative prehospital anaesthesia case number

With physicians’ increasing experience, 30-day mortality decreased (Fig. 3A). The crude mortality decreased from 38 to 26% from the lowest to highest experience groups. After adjusting for patient-level factors, the multivariate logistic regression analysis showed the same association with an odds ratio for 30-day mortality of the highest experience group of 0.59 (95% CI 0.38–0.94, lowest experience group as a reference, Fig. 3B). The Hosmer–Lemeshow test indicated a good fit for the model (*P* = 0.87), while Nagelkerke’s *R*^*2*^ value was 35.6%. The detailed results of the logistic regression model are in Supplement 2. Physicians’ cumulative PHAAM experience was associated with 30-day mortality across all three sensitivity analyses (Supplement 1, Tables A–C).

## Discussion

As far as we know, this is the first paper addressing the PHAAM learning curve of newly recruited physicians in a prehospital critical care setting. We demonstrated that the quality of prehospital anaesthesia provided was good, reflecting high competence of baseline skills. However, we found that combining neuromuscular blocking, sedative agents and mechanical ventilation increased whilst OST decreased as experience in PHAAM was accumulated. We also found a decrease in 30-day mortality with higher experience. The reasons for these findings are likely multifactorial.

Expectations for the quality of advanced prehospital care in physician-led systems have increased during the last decades [16]. Furthermore, the culture surrounding patient safety has steadily evolved, and many services nowadays expect PHAAM to meet the same standards as in-hospital airway management [17]. Organisations, especially in Europe, have employed experienced medical professionals in prehospital services to ensure high standards in advanced prehospital care. Thus, the responsibility of HEMS organisations is to ensure adequate clinical, operational, and non-technical skills of personnel. So far, no data have been published regarding the learning curve of new physicians beginning a career in HEMS. The turnover time of physicians varies between services, and the effect on the patient outcome or management by a new recruit compared to a senior HEMS physician is unknown.

The quality of prehospital critical care is challenging to measure. The expert consensus-based quality indicators for PHAAM have been published recently [8]. All available process- and outcome-related indicators were included in the current study. We consider the selection of the outcome variables mostly adequate. However, due to some data being unavailable in the HEMS registry, this study does not cover all spectrums of quality. For example, information on overall intubation experience could not be assessed as we had no data on in-hospital intubations. Furthermore, the mortality rate is a crude outcome measure, and the quality of prehospital anaesthesia and physiological stabilisation may more likely affect the functional outcome or quality of life. Data on these are difficult to collect routinely; thus, such data are still rarely reported in prehospital studies.

Skill development in tracheal intubation has been studied in a controlled environment in several studies. However, the duration of these studies remains limited, and few studies report the amount of cumulative experience needed to achieve an FPS exceeding 80% [7]. Several studies have shown that emergency airway management incurs considerably higher risk, and complications are more common when two or more attempts are needed [18, 19]. We found no studies on how physicians already well-versed in elective anaesthesia develop skills in emergency airway management.

We observed high FPS rates in all physician groups, and the rates, although confidence intervals overlapped, increased consistently as physicians’ exposure to prehospital anaesthesia increased. A systematic review and meta-analysis by Crewdson et al. compared prehospital intubation’s success by different providers. Across the 14 studies reporting FPS, the overall rate was 78% amongst all providers and 87% (77–98) for physicians – slightly below our results [20]. We also observed that the first endotracheal intubation FPS rates were slightly higher than the successive 11–20 attempts, perhaps because the first prehospital endotracheal intubations were performed during training under a more experienced colleague’s supervision.

Our previous paper demonstrated an association between the frequency of a physician’s prehospital anaesthesia cases and 30-day mortality [4]. In our studies, merely the rate of complications or technical success was insufficient to explain the difference in mortality. We suspect one key factor underlying the differences in outcomes is non-technical skill development. The prehospital setting vastly differs from the in-hospital environment. Sufficient and frequent experience enables physicians to better utilise prehospital resources and make better decisions regarding treatment, transport, and patient safety. The shorter OST in the groups with higher experience demonstrated somewhat better efficiency.

Considering our current study alongside our previous findings, cumulative experience and frequent repetition appear vital for delivering the highest quality care. While these factors are interrelated, we believe both should be evaluated independently when planning how new physicians develop and maintain proficiency in PHAAM. We hypothesise that performance declines more gradually with high cumulative experience and that the interval between repetitions influences how performance improves during the initial stages of skill development.

A larger study would be required to assess if physicians with similar cumulative experience gained over varying timeframes achieve different outcomes. Combining data from several prehospital airway registries may answer these questions, helping optimise the orientation and skill maintenance of HEMS physicians. Wide adoption of the published quality indicators for PHAAM would considerably assist in these kinds of studies [8].

This study has several strengths. The database used as a source includes all HEMS missions in the country during the study period [10]. The study’s length allowed sufficient time to observe the development of practice over several years; thus, the number of cases was relatively high. However, we also identified some limitations. One in five patients had post-intubation blood pressure and oxygen saturation missing, which is quite high. Furthermore, the data may not be missing randomly; patients in a more critical condition may be more likely to have missing values, which could bias the results. A comparison of patients with these values missing versus those not missing can be found in a separate online supplement (Supplement 3B-C).

Due to the data being anonymised, we could not obtain detailed information on individual physicians’ prior anaesthesia experience, nor did we have data about their airway management outside HEMS. All vital signs are manually recorded into the database, making typing errors possible. Also, protocol does not dictate which exact values should be entered for vital signs at the time of patient encounter, post-intubation and at handover. Therefore, physicians may unconsciously be biased in recording better values in the database when several choices are available. For example, a physician may dismiss low oxygen saturation due to poor signal quality.

Misreporting whether a patient received airway management is possible, but we believe this is uncommon. Since details are noted shortly after the mission with mission records available, we consider the low likelihood of recall or reporting bias to be a strength of our study.

Physicians are allowed to deny missions when they believe HEMS provides no additional benefit. Consequently, if the threshold for participation changes with experience, selection bias might create an illusion of improved outcomes. However, the studied patients were comparable between groups. This study was inadequately powered to study specific patient categories separately. The patients undergoing prehospital anaesthesia form a highly heterogenic group; thus, the controlled variables may affect survival differently depending on the patients’ condition.

During the study period, there may have been general improvements across the board in prehospital and in-hospital management, which may have enhanced outcomes towards the end of the study period. We lack data on in-hospital treatment, which represents another limitation. Between 2015 and 2016, two of the five physician-staffed HEMS bases implemented a standard operating procedure (SOP) for PHAAM. In a separate study, we noted a decrease in OST and an improvement in FPS following the implementation of the SOP, although no improvement in 30-day mortality was observed [21]. Some improvements seen in our study may have been mitigated by implementing the SOP or adopting best practices based on it.

As EMSs among countries differ markedly, these results must be cautiously generalised. These results should be adaptable to European HEMS systems staffed by physicians with substantial anaesthesia experience. Our results may not be generalisable to HEMS units staffed mainly by non-anaesthesiologists or paramedics. In HEMS systems, where case volume is much lower than in our services, the results may not apply. Our study concentrated only on physicians’ experience in PHAAM. As prehospital critical care is teamwork, the other team members’ experience may also contribute. For example, a HEMS physician’s inexperience may be compensated by the experience of other team members.

### Clinical significance

Our findings indicate that physicians recruited to the HEMS provide good-quality PHAAM. Although a solid foundation in advanced airway management is a prerequisite for employment, significant improvement occurs even after multiple years and cumulative cases of PHAAM. Consequently, we recommend limiting the turnover of HEMS physicians to allow expertise development in prehospital anaesthesia management. Regular shifts at the beginning of the HEMS career are likely important even for consultant anaesthesiologists. If the overall exposure to PHAAM is low in the HEMS base, periodic rotations in high-volume areas might be advantageous.

## Conclusions

In a prehospital critical care service, newly recruited physicians with substantial anaesthesia experience had a great overall quality of PHAAM, measured with the rate of FPS and the presence of physiological derangement during anaesthesia. Improvement was seen even after a high number of cases. The cumulative experience in PHAAM was associated with lower 30-day mortality. Limiting physician turnover may improve the system’s quality.

## Supplementary Information

Supplementary Material 1.

Supplementary Material 2.

Supplementary Material 3.

## Data Availability

No datasets were generated or analysed during the current study.

## Abbreviations

EMS: Emergency medical services
FPS: First-pass success rate
GCS: Glasgow Coma Score
HEMS: Helicopter emergency medical services
IQR: Interquartile range
OST: On-scene time
PHAAM: Prehospital advanced airway management
PIN: Personal identification number
SpO_2_: Blood oxygenation saturation
STROBE: Strengthening the reporting of observational studies in epidemiology
TBI: Traumatic brain injury
