# Supplementary material for: Association of mortality and physician experience in prehospital anaesthesia: a registry study on new physicians in Finnish helicopter emergency medical services
Source: Scand J Trauma Resusc Emerg Med. 2025 May 30;33:98. doi: 10.1186/s13049-025-01412-4 (PMC12125928; doi:10.1186/s13049-025-01412-4)
Supplement: Supplementary file 1 — Supplementary Material 1. [file 13049_2025_1412_MOESM1_ESM.docx]

**Supplement 1.** Supplemental methods and sensitivity analyses

**Sample size**

No power calculation was performed; all available data were used instead.

**Statistical methods**

Small patient categories were combined with 'Other' to simplify to six main categories, preventing small groups that could yield unpredictable results when combined with multivariate logistic regression analysis. Additionally, 'Stroke' was merged with 'Acute neurology, excluding stroke' due to significant overlap and misclassification noted in prior research. [1] As Finnish HEMS does not respond to psychiatric emergencies, this group was labelled 'Intoxication'. [1]. For the rare cases where patients were sedated before HEMS arrival, GCS was analysed as a 3, as these were labelled as “sedated” without a specific value in the database.

**Sensitivity analyses**

We performed three sensitivity analyses to test the robustness of our findings. First, we excluded all patients categorised as intoxications by the physician. This was done to exclude confounding by this group as our previous studies have shown Finnish HEMS treat these patients quite frequently compared to similar services and they have shown to have high survival rates.[2,3]  Second, we excluded all patients from the HEMS unit serving the region surrounding the capital. This unit manages the highest number of patients, and they are, on average, slightly younger and closer to receiving hospitals compared to patients from other units. We performed this exclusion to ensure results are generalisable beyond this particular setting. Third, we analysed the physicians’ cumulative prehospital anesthesia experience as a continuous variable using a natural logarithm of the number of cases.

1. Tommila M, Pappinen J, Raatiniemi L, Saviluoto A, Toivonen T, Björkman J, et al. Standardised data collection in prehospital critical care: a comparison of medical problem categories and discharge diagnoses. Scand J Trauma, Resusc Emerg Med. 2022;30:26.

2. Saviluoto A, Björkman J, Olkinuora A, Virkkunen I, Kirves H, Setälä P, et al. The first seven years of nationally organized helicopter emergency medical services in Finland - the data from quality registry. Scand J Trauma Resusc Emerg Medicine. 2020;28:46.

3. Björkman J, Laukkanen-Nevala P, Olkinuora A, Pulkkinen I, Nurmi J. Short-term and long-term survival in critical patients treated by helicopter emergency medical services in Finland: a registry study of 36 715 patients. Bmj Open. 2021;11:e045642.

**Supplemental Table A.** Sensitivity analysis. Results of logistic regression model for 30-day mortality after prehospital anaesthesia excluding all 247 patients categorised as “Intoxication” by physician.

| **Characteristic** | **OR** | **95% CI** | **p-value** |
| --- | --- | --- | --- |
| Age (years) | 1.04 | 1.03, 1.05 | **<0.001** |
| Gender |  |  |  |
| Female | — | — |  |
| Male | 1.15 | 0.86, 1.52 | 0.3 |
| Patient category |  |  |  |
| Trauma | — | — |  |
| Out-of-hospital cardiac arrest | 0.73 | 0.47, 1.15 | 0.2 |
| Neurological | 0.79 | 0.56, 1.12 | 0.2 |
| Other | 0.27 | 0.15, 0.46 | **<0.001** |
| Time from alarm to patient (min) | 1.00 | 1.00, 1.01 | 0.3 |
| Heart rate at patient encounter (/min) | 0.99 | 0.99, 1.00 | **0.012** |
| Systolic blood pressure (mmHg) | 1.01 | 1.00, 1.01 | **<0.001** |
| Glasgow Coma Score at patient encounter | 0.85 | 0.81, 0.89 | **<0.001** |
| Oxygen saturation at patient encounter (%) | 0.96 | 0.95, 0.98 | **<0.001** |
| Transported to university hospital | 0.91 | 0.52, 1.59 | 0.7 |
| Cumulative number of prehospital anaesthesia cases by physician |  |  |  |
| 1–10 | — | — |  |
| 11–20 | 0.88 | 0.57, 1.36 | 0.6 |
| 21–40 | 0.83 | 0.57, 1.22 | 0.4 |
| 41–80 | 0.83 | 0.56, 1.22 | 0.3 |
| >80 | 0.60 | 0.38, 0.96 | **0.034** |

n = 1 391

**Supplemental Table B.** Sensitivity analysis. Results of logistic regression model for 30-day mortality after prehospital anaesthesia excluding all 693 patients from the helicopter emergency medical services unit with highest number of missions.

| **Characteristic** | **OR** | **95% CI** | **p-value** |
| --- | --- | --- | --- |
| Age (years) | 1.04 | 1.03, 1.05 | **<0.001** |
| Gender |  |  |  |
| Female | — | — |  |
| Male | 1.05 | 0.74, 1.50 | 0.8 |
| Patient category |  |  |  |
| Trauma | — | — |  |
| Out-of-hospital cardiac arrest | 0.70 | 0.39, 1.24 | 0.2 |
| Neurological | 0.79 | 0.51, 1.22 | 0.3 |
| Intoxication | 0.08 | 0.02, 0.22 | **<0.001** |
| Other | 0.28 | 0.14, 0.52 | **<0.001** |
| Time from alarm to patient (min) | 1.00 | 0.99, 1.01 | 0.6 |
| Heart rate at patient encounter (/min) | 0.99 | 0.98, 1.00 | **0.001** |
| Systolic blood pressure (mmHg) | 1.00 | 1.00, 1.01 | **0.038** |
| Glasgow Coma Score at patient encounter | 0.88 | 0.83, 0.93 | **<0.001** |
| Oxygen saturation at patient encounter (%) | 0.96 | 0.94, 0.98 | **<0.001** |
| Transported to university hospital | 1.10 | 0.38, 3.39 | 0.9 |
| Cumulative number of prehospital anaesthesia cases by physician |  |  |  |
| 1–10 | — | — |  |
| 11–20 | 0.91 | 0.54, 1.51 | 0.7 |
| 21–40 | 0.80 | 0.49, 1.28 | 0.3 |
| 41–80 | 0.75 | 0.47, 1.20 | 0.2 |
| >80 | 0.52 | 0.27, 0.96 | **0.041** |

n = 945

**Supplemental Table C.** Sensitivity analysis. Results of logistic regression model for 30-day mortality after prehospital anaesthesia. Physicians’ prehospital anaesthesia experience expressed as the natural logarithm of the number of previous cases by the physician (Ln n)

| **Characteristic** | **OR** | **95% CI** | **p-value** |
| --- | --- | --- | --- |
| Age (years) | 1.04 | 1.03, 1.05 | **<0.001** |
| Gender (Female) | 0.88 | 0.67, 1.17 | 0.4 |
| Patient category |  |  |  |
| Trauma | — | — |  |
| Out-of-hospital cardiac arrest | 0.75 | 0.48, 1.18 | 0.2 |
| Neurological | 0.82 | 0.58, 1.16 | 0.3 |
| Intoxication | 0.11 | 0.05, 0.22 | **<0.001** |
| Other | 0.26 | 0.15, 0.45 | **<0.001** |
| Time from alarm to patient (min) | 1.01 | 1.00, 1.01 | 0.2 |
| Heart rate at patient encounter (/min) | 0.99 | 0.99, 1.00 | **0.016** |
| Systolic blood pressure (mmHg) | 1.01 | 1.00, 1.01 | **<0.001** |
| Glasgow Coma Score at patient encounter | 0.86 | 0.82, 0.90 | **<0.001** |
| Oxygen saturation at patient encounter (%) | 0.96 | 0.95, 0.98 | **<0.001** |
| Transported to university hospital | 0.93 | 0.54, 1.61 | 0.8 |
| Natural logarithm of the cumulative number of prehospital anaesthesia cases by physician  n = 1 638 | 0.88 | 0.79, 0.99 | **0.039** |
