# Supplementary material for: Association of mortality and physician experience in prehospital anaesthesia: a registry study on new physicians in Finnish helicopter emergency medical services
Source: Scand J Trauma Resusc Emerg Med. 2025 May 30;33:98. doi: 10.1186/s13049-025-01412-4 (PMC12125928; doi:10.1186/s13049-025-01412-4)
Supplement: Supplementary file 2 — Supplementary Material 2. [file 13049_2025_1412_MOESM2_ESM.docx]

**Supplement 2.** Results of logistic regression model for 30-day mortality after prehospital anaesthesia.

|  | B | S.E. | Sig. | Odds ratio | 95% confidence interval of odds ratio | |
| --- | --- | --- | --- | --- | --- | --- |
|  |  |  |  |  | Lower | Upper |
| Age, years | 0,041 | 0,004 | 0,000 | 1,041 | 1,033 | 1,050 |
| Sex, female | -0,179 | 0,142 | 0,207 | 0,836 | 0,633 | 1,104 |
| Systolic blood pressure, mmHg | 0,007 | 0,002 | 0,000 | 1,007 | 1,003 | 1,010 |
| Heart rate, min^-1^ | -0,006 | 0,003 | 0,029 | 0,994 | 0,989 | 0,999 |
| Oxygen saturation | -0,038 | 0,009 | 0,000 | 0,963 | 0,947 | 0,979 |
| Glasgow Coma Score | -0,157 | 0,025 | 0,000 | 0,855 | 0,813 | 0,898 |
| Delay from alarm to patient, min | 0,005 | 0,004 | 0,269 | 1,005 | 0,996 | 1,013 |
| University hospital | -0,083 | 0,275 | 0,762 | 0,920 | 0,536 | 1,578 |
|  |  |  |  |  |  |  |
| Patient category |  |  |  |  |  |  |
| Trauma (reference) |  |  | 0,000 |  |  |  |
| Out-of-hospital cardiac arrest | -0,310 | 0,227 | 0,173 | 0,734 | 0,470 | 1,145 |
| Neurological | -0,230 | 0,176 | 0,192 | 0,795 | 0,563 | 1,123 |
| Intoxication | -2,307 | 0,401 | 0,000 | 0,100 | 0,045 | 0,218 |
| Other | -1,344 | 0,284 | 0,000 | 0,261 | 0,150 | 0,455 |
|  |  |  |  |  |  |  |
| Cumulative number of prehospital anaesthesia cases by the physician |  |  |  |  |  |  |
| 1-10 (reference) |  |  | 0,281 |  |  |  |
| 11-20 | -0,124 | 0,218 | 0,569 | 0,883 | 0,577 | 1,353 |
| 21-40 | -0,157 | 0,192 | 0,415 | 0,855 | 0,586 | 1,246 |
| 41-80 | -0,162 | 0,197 | 0,409 | 0,850 | 0,578 | 1,251 |
| >80 | -0,522 | 0,234 | 0,026 | 0,594 | 0,375 | 0,939 |
|  |  |  |  |  |  |  |
| Constant | 1,377 | 0,995 | 0,166 | 3,961 |  |  |
