# Supplementary material for: Association of mortality and physician experience in prehospital anaesthesia: a registry study on new physicians in Finnish helicopter emergency medical services
Source: Scand J Trauma Resusc Emerg Med. 2025 May 30;33:98. doi: 10.1186/s13049-025-01412-4 (PMC12125928; doi:10.1186/s13049-025-01412-4)
Supplement: Supplementary file 3 — Supplementary Material 3. [file 13049_2025_1412_MOESM3_ESM.docx]

**Supplement 3A.** Missing data

| **Variable** | **Missing n (%)** |
| --- | --- |
| Age | 0 (0) |
| Gender | 6 (0.4) |
| Patient category | 0 (0) |
| Glasgow Coma Score at patient encounter | 0 (0) |
| Systolic blood pressure at patient encounter | 66 (4.0) |
| Oxygen saturation at patient encounter | 103 (6.3) |
| Oxygen saturation post-intubation | 333 (20) |
| Oxygen saturation at handover | 122 (7.4) |
| End-Tidal carbon dioxide at handover | 87 (5.3) |
| Mechanical ventilation | 0 (0) |
| Anaesthetic & neuromusclular blocking agent used | 0 (0) |
| Blood pressure post-intubation | 334 (20) |
| Blood pressure at handover | 110 (6.7) |
| Heart rate at patient encounter | 25 (1.5) |
| Time from alarm to patient | 0 (0) |
| Transported to university hospital | 18 (1.1) |
| Cumulative number of prehospital anaesthesia cases by physician | 0 (0) |
| 30-day mortality | 69 (4.2) |

**Supplement 3B.** Comparison of patients with missing vs. not missing post-intubation oxygen saturation. Presented as median (interquartile range) or n (%).

| **Characteristic** | **Missing**, n = 333 | **Not missing**, n = 1,305 |
| --- | --- | --- |
| Age (years) | 64 (49–73) | 57 (35–70) |
| Gender |  |  |
| Female | 228 (69%) | 817 (63%) |
| Male | 102 (31%) | 485 (37%) |
| Patient category |  |  |
| Trauma | 50 (15%) | 363 (28%) |
| Out-of-hospital cardiac arrest | 217 (65%) | 12 (0.9%) |
| Neurological | 31 (9.3%) | 551 (42%) |
| Intoxication | 16 (4.8%) | 231 (18%) |
| Other | 19 (5.7%) | 148 (11%) |
| Time from alarm to patient (min) | 27 (18–40) | 23 (17–34) |
| Heart rate at patient encounter (/min) | 96 (79–115) | 92 (75–113) |
| Systolic blood pressure at patient encounter (mmHg) | 127 (108–150) | 133 (110–164) |
| Glasgow Coma Score at patient encounter | 3.0 (3.0–5.0) | 4.0 (3.0–7.0) |
| Oxygen saturation at patient encounter (%) | 97 (91–99) | 97 (92–99) |
| Transported to university hospital | 286 (89%) | 1,227 (95%) |
| Cumulative number of prehospital anaesthesia cases by physician |  |  |
| 1–10 | 104 (31%) | 223 (17%) |
| 11–20 | 50 (15%) | 206 (16%) |
| 21–40 | 64 (19%) | 334 (26%) |
| 41–80 | 72 (22%) | 333 (26%) |
| >80 | 43 (13%) | 209 (16%) |
| Oxygen saturation at handover | 98.00 (95.00–99.00) | 99.00 (97.00–100.00) |
| End-Tidal CO2 at handover | 4.30 (4.00–4.70) | 4.40 (4.00–4.80) |
| Mechanical ventilation | 275 (83%) | 1,096 (84%) |
| Anaesthetic & neuromusclular blocking agent used | 265 (80%) | 1,085 (83%) |
| Blood pressure post-intubation | 83 (67–122) | 124 (102–148) |
| Blood pressure at handover | 122 (110–140) | 128 (110–143) |
| 30-day mortality | 140 (43%) | 364 (29%) |

**Supplement 2C.** Comparison of patients with missing vs. not missing post-intubation blood pressure. Presented as median (interquartile range) or n (%).

| **Characteristic** | **Missing**, n = 334 | **Not missing**, n = 1,304 |
| --- | --- | --- |
| Age (years) | 64 (50–74) | 57 (35–70) |
| Gender |  |  |
| Female | 228 (69%) | 817 (63%) |
| Male | 103 (31%) | 484 (37%) |
| Patient category |  |  |
| Trauma | 49 (15%) | 364 (28%) |
| Out-of-hospital cardiac arrest | 217 (65%) | 12 (0.9%) |
| Neurological | 33 (9.9%) | 549 (42%) |
| Intoxication | 14 (4.2%) | 233 (18%) |
| Other | 21 (6.3%) | 146 (11%) |
| Time from alarm to patient (min) | 27 (18–40) | 23 (17–34) |
| Heart rate at patient encounter (/min) | 97 (79–115) | 92 (75–113) |
| Systolic blood pressure at patient encounter (mmHg) | 129 (108–150) | 133 (110–164) |
| Glasgow Coma Score at patient encounter | 3.0 (3.0–5.0) | 4.0 (3.0–7.0) |
| Oxygen saturation at patient encounter (%) | 97 (90–99) | 97 (92–99) |
| Transported to university hospital | 288 (89%) | 1,225 (94%) |
| Cumulative number of prehospital anaesthesia cases by physician |  |  |
| 1–10 | 103 (31%) | 224 (17%) |
| 11–20 | 50 (15%) | 206 (16%) |
| 21–40 | 63 (19%) | 335 (26%) |
| 41–80 | 75 (22%) | 330 (25%) |
| >80 | 43 (13%) | 209 (16%) |
| Oxygen saturation post-intubation | 95.0 (92.0–99.0) | 99.0 (97.0–100.0) |
| Oxygen saturation at handover | 98.00 (95.00–99.00) | 99.00 (97.00–100.00) |
| End-Tidal CO2 at handover | 4.30 (4.00–4.70) | 4.40 (4.00–4.80) |
| Mechanical ventilation | 277 (83%) | 1,094 (84%) |
| Anaesthetic & neuromusclular blocking agent used | 265 (79%) | 1,085 (83%) |
| Blood pressure at handover | 122 (110–140) | 127 (110–142) |
| 30-day mortality | 138 (42%) | 366 (29%) |
